# Coupling treatment planning with navigation system: a new technological approach in treatment of head and neck tumors by electrochemotherapy

**DOI:** 10.1186/1475-925X-14-S3-S2

**Published:** 2015-08-27

**Authors:** Ales Groselj, Bor Kos, Maja Cemazar, Jure Urbancic, Grega Kragelj, Masa Bosnjak, Biserka Veberic, Primoz Strojan, Damijan Miklavcic, Gregor Sersa

**Affiliations:** 1 Clinic of Otorhinolaryngology and Cervicofacial Surgery, University Medical Centre Ljubljana, Zaloska 2, Ljubljana SI-1000, Slovenia; 2 Department of Biomedical Engineering, Faculty of Electrical Engineering, University of Ljubljana, Trzaska 25, Ljubljana SI-1000, Slovenia; 3 Department of Experimental Oncology, Institute of Oncology Ljubljana, Zaloska 2, Ljubljana SI-1000, Slovenia; 4 Department of Radiotherapy, Institute of Oncology Ljubljana, Zaloska 2, Ljubljana SI-1000, Slovenia

**Keywords:** electrochemotherapy, head and neck tumors, squamous cell carcinoma, treatment planning, navigation system

## Abstract

**Background:**

Electrochemotherapy provides highly effective local treatment for a variety of tumors. In deep-seated tumors of the head and neck, due to complex anatomy of the region or inability to cover the whole tumor with standard electrodes, the use of long single needle electrodes is mandatory. In such cases, a treatment plan provides the information on the optimal configuration of the electrodes to adequately cover the tumor with electric field, while the accurate placement of the electrodes in the surgical room in patients can remain a problem. Therefore, during electrochemotherapy of two head and neck lymph-node metastases of squamous cell carcinoma origin, a navigation system for placement of electrodes was used.

**Patient and methods:**

Electrochemotherapy of two lymph-node metastases of cutaneous squamous cell carcinoma, one in the left parotid gland and the other in the neck just behind the left mandibular angle, was performed using intravenous administration of bleomycin and long single needle electrodes. The tumors were treated according to the prepared treatment plan, and executed with the use of navigation system.

**Results:**

Coupling of treatment plan with the navigation system aided to an accurate placement of the electrodes. The navigation system helped the surgeon to identify the exact location of the tumors, and helped with the positioning of the long needle electrodes during their insertion, according to treatment plan. Five electrodes were inserted for each metastasis, one centrally in the tumor and four in the periphery of the tumor. Five weeks after electrochemotherapy, computed tomography images demonstrated partial response of the first metastasis and complete response of the second one. Six weeks after electrochemotherapy, fine-needle aspiration biopsy specimen obtained from the treated lesions revealed necrosis and inflammatory cells, without any viable tumor cells.

**Conclusion:**

We describe a new technological approach for electrochemotherapy of deep-seated head and neck tumors, coupling of the treatment planning with navigation system for accurate placement of the single long needle electrodes into and around the tumors, according to the treatment plan. Evidence of its effectiveness on two lymph-node metastases of cutaneous squamous cell carcinoma origin in neck lymph is provided.

## Background

Electroporation based technology for biomedical applications is quickly developing [1]. Using different electroporation protocols it can be used for tumor ablation (irreversible electroporation [2-8], nanopulses [9]), for gene transfer to cells i.e. gene electrotransfer [10], and delivery of drugs i.e. electrochemotherapy [11,12]. Electrochemotherapy uses electroporation for increased drug delivery to tumors and its effectiveness has been demonstrated in a large variety of tumors [13], predominantly for the treatment of cutaneous tumors using electrodes with fixed geometry [12,14]. For the treatment of deep seated tumors, single long needle electrodes were developed, that can also be placed in an irregular pattern in order to cover irregularly shaped tumors larger than 2 cm in diameter [15]. With appropriate imaging support, this approach also enables appropriate placement of the electrodes with respect to sensitive structures such as major vessels and nerve bundles. The applicability of this approach has already been demonstrated and verified in the treatment of liver metastases, where antitumor effectiveness of electrochemotherapy was confirmed also in tumors located in close proximity or in-between the major vessels [16,17].

A treatment planning method has been developed for the treatment of deep seated tumors [18,19]. It has been evolving through the experience in treatment of liver metastases [16,17], and now also a web based application is under development (http://www.visifield.com). The aim of this method is to prepare treatment plans consisting of instructions for positioning of electrodes and the voltages to be applied to each electrode pair, which should ensure a successful treatment. Briefly, the treatment plan is prepared using Computed Tomography (CT) or Magnetic Resonance Imaging (MRI) based tumor images, which are used for segmentation of the tumor and important normal structures in its surrounding. The electric field distribution is computed taking into account different tissue conductivities [20] and changes in conductivity due to electroporation [21]. Adequate coverage of the tumor (i.e. target) is assured with optimized electrode position and voltages. However, deviations in implementation of the treatment plan can occur during the treatment in the clinic, because the exact position of the tumor inside the body and in its relation to the neighboring structures *i*) is different compared to the tumor position during treatment plan creation; or *ii*) cannot be determined with sufficient precision. In both instances, spatial relationship of the electrodes to the treated tumor does not correspond to the treatment plan and electric field coverage of the tumor is suboptimal. Consequently, the treatment effectiveness could be seriously hampered and toxicity increased [22]. Treatment effectiveness could be improved with the aid of the existing techniques that enable exact positioning of the tumor and electrodes in the tissue during the clinical intervention.

In image-guided surgery, navigation system is used as assistance to display real-time data on tumor position in relation to the preoperative CT or MR scans of a patient. It has been successfully implemented in otorhinolaryngologic surgery as a tool to access difficult anatomic areas and for stereotactic biopsy procedures.

With respect to effectiveness of electrochemotherapy in cutaneous tumors, clinical results gained in the group of tumors of the head and neck region is less promising [13,23,24]. Possible explanations for the worse outcome are: deep seated parts of these tumors exist, hidden under the visible skin or mucosal surface and of considerable volume, head and neck tumors typically have irregular shape, and finally, the size of these tumors can be up to 10 cm in diameter. For such tumors, electrodes with fixed geometry are not suitable, because they cannot be inserted deep enough, to reach the deep margins of these tumors. Thus, the use of single long needle electrodes is indicated in such cases [15]. When using long needle electrodes, treatment planning with visualization of electric field distribution and coverage of the tumor can offer a significant advantage over blind insertion of the needles. Furthermore, coupling of the treatment plan with navigation system improves precision of electrode placement during the procedure and provides a technological advancement in the treatment of deep seated tumors in the head and neck region.

The aim of our study was to couple treatment planning with a navigation system as a new technological approach in treatment of head and neck tumors by electrochemotherapy. The feasibility and effectiveness of this concept was demonstrated in the case of a patient with two lymphatic metastases in the region of the head and neck.

## Patient and methods

### Patient characteristics

An 88-year-old male patient with a history of several surgical procedures for squamous and basal cell skin cancers was treated at the Department of Otorhinolaryngology and Cervicofacial Surgery, University Medical Centre Ljubljana, Ljubljana, Slovenia. The patient had previously been irradiated due inoperable lymphatic metastasis of squamous cell carcinoma origin, located in the left parotid region. Tumor was 42 mm in diameter, deeply infiltrated into the left carotid space and jugular vein. Complete response of the tumor was achieved after a cumulative dose of 70 Gy delivered by 6 MV linear accelerator photon beam in 2 Gy daily fractions.

Eleven months after radiotherapy a new lesion was clinically detected behind the left mandibular angle. In addition, diagnostic workup also revealed disease recurrence in the deep lobe of the left parotid gland. On CT scans, diameters of the two lesions were 20.3 mm (left parotid gland, metastasis No. 1) and a 20.6 mm (centrally necrotic lymph node behind the left mandibular angle, metastasis No. 2). Fine needle aspiration biopsy confirmed metastases of squamous cell carcinoma. The patient was offered electrochemotherapy as the only potentially curative treatment option, and after detailed information about its advantages and drawbacks he signed informed consent for treatment and publication of the data. The study was approved by the National Ethics Committee 182/02/14.

### Treatment plan

**Figure 1:**
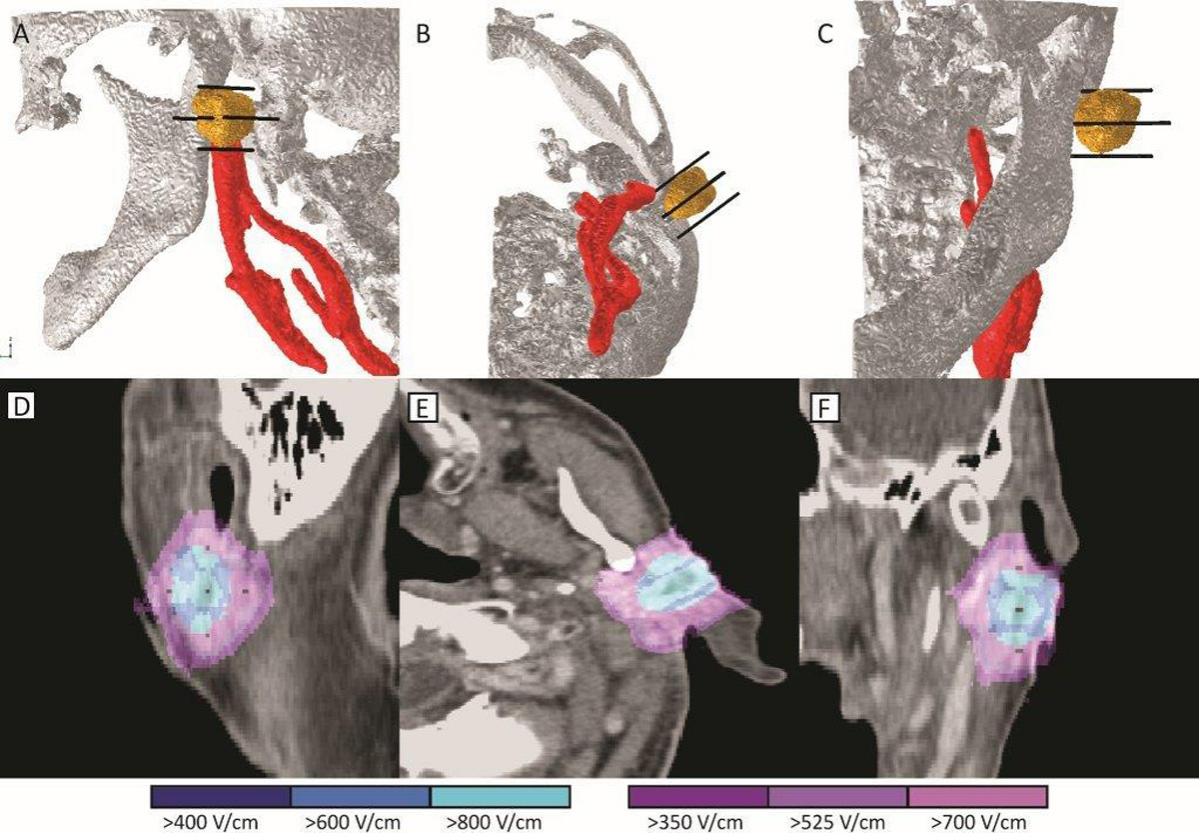
**Treatment plan for parotid metastasis (No.1)**. The upper row (A-C) shows the 3-D model with the bone (white), tumor (yellow), blood vessels (red), and electrodes (black) shown for clarity. Lower row (D-F) shows the electric field strength according to the treatment plan. Blue color indicates tumor covered above 400 V/cm, while violet indicates normal tissue above 350 V/cm. Care was taken to ensure electrodes would avoid the major blood vessels and the bone.

**Figure 2:**
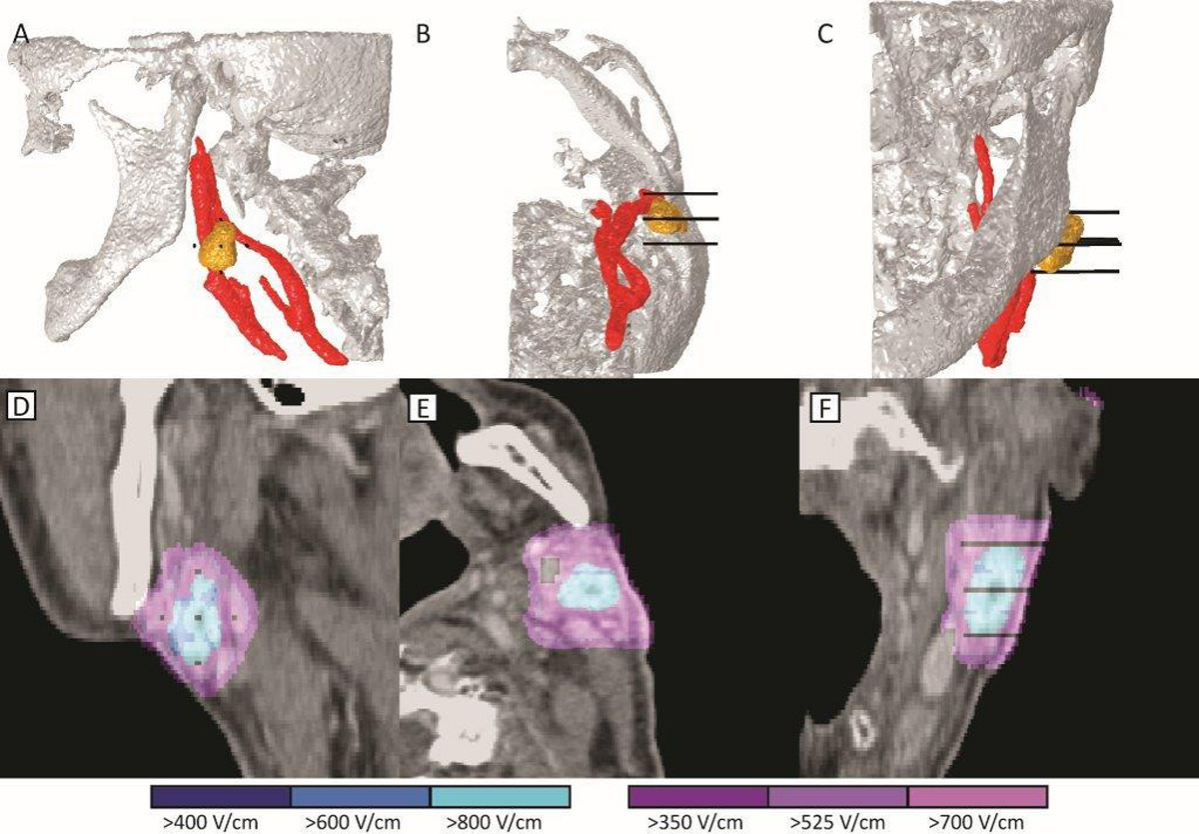
**Treatment plan for metastasis in the neck (No.2) behind the left mandibular angle**. The upper row (A-C) shows the 3-D model with the bone (white), tumor (yellow), blood vessels (red), and electrodes (black). Lower row (D-F) shows the electric field strength according to the treatment plan. Blue color indicates tumor covered above 400 V/cm, while violet indicates normal tissue above 350 V/cm. Care was taken to avoid penetration of the electrodes into the blood vessels.

**Figure 3:**
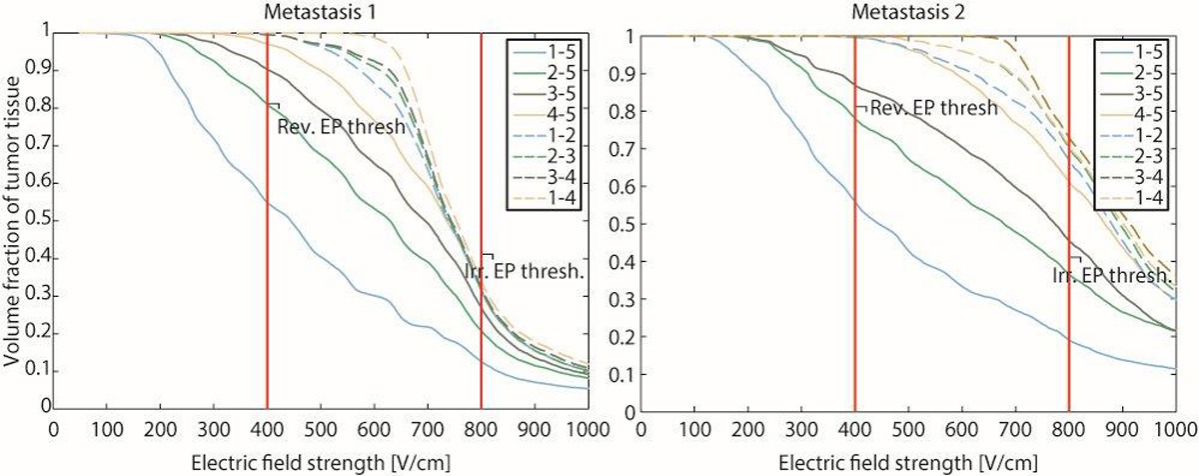
**Cumulative coverage of both metastases with electric fields after each electrode pair**. The graphs show the volume fraction of tumor tissue that is covered with electric field of at least the strength indicated on the horizontal axis. For visual reference, reversible and irreversible electroporation thresholds for 8 pulses of 100 µs are indicated.

A treatment plan was prepared for both metastases. The bone and both tumor lesions were segmented using the online Visifield tool (http://www.visifield.com). The blood vessels were segmented using the 3-D adaptive contour implemented in ITK-SNAP (http://www.itksnap.org) [25]. Five electrodes were chosen as an optimal solution for this clinical situation: one central electrode was placed in the metastasis and four additional electrodes around each of the tumors in surrounding healthy tissue. Care was taken to ensure that the belt of normal tissue up to 5 mm wide around the node (i.e. a safety margin) was also covered with an electric field and that the electrodes did not penetrate the blood vessels or into the bone. The treatment plan was optimized using procedures described previously [26-29]. The primary aim was to ensure coverage of the whole tumor and safety margin with electric fields above the reversible electroporation threshold [29], while also ensuring adequate robustness. Robustness here indicates that the coverage of the tumor with sufficient fields would not be lowered, given small uncertainties in the tissue parameters and errors in electrode positioning [22]. The robustness of the treatment in our case was established by creation of the safety margins as shown on Figures 1 and 2, and by appropriate coverage of the whole tumor volume with electric fields shown on Figure 3. The coverage of the target tumor is slightly above the minimum required, but this is to a certain degree desirable, since electroporation threshold has the largest effect on robustness of the treatment [22]. The electric field distribution in tumor was visualized in the Visifield tool to verify the coverage of the target volume. The treatment plan and electric field distribution are shown in Figures 1 and 2 for metastasis No. 1 and No. 2, respectively.

The tips and entry points of the 5 individual needles were marked on original CT images, by setting the corresponding pixel intensity values to 3000. The same DICOM images were then imported into the navigation system, and the marked points were located on the images and used to position the guiding vector of the navigation system. The same images were used for the treatment planning; the interval between image acquisition and treatment was 27 days. In order to keep the minimal error, it is recommended to keep the time interval as short as possible, since in the meantime the tumor may grow and change its shape as well as the shape and position of the neighboring structures.

### Navigation system

Optical navigation system Colibri (Brainlab AG, Feldkirchen, Germany) with ENT V2.1.1 software package was used. The system itself is capable of navigation within less than 1 mm accuracy; built-in tolerance in registration with system reported good precision is 1 mm. After the registration confirming known anatomical points on patient and in navigation, a test of precision is made by the surgeon and is demanded by system software. In case of discrepancy of more than 1 mm re-registration is done until expected accuracy is confirmed.

**Figure 4:**
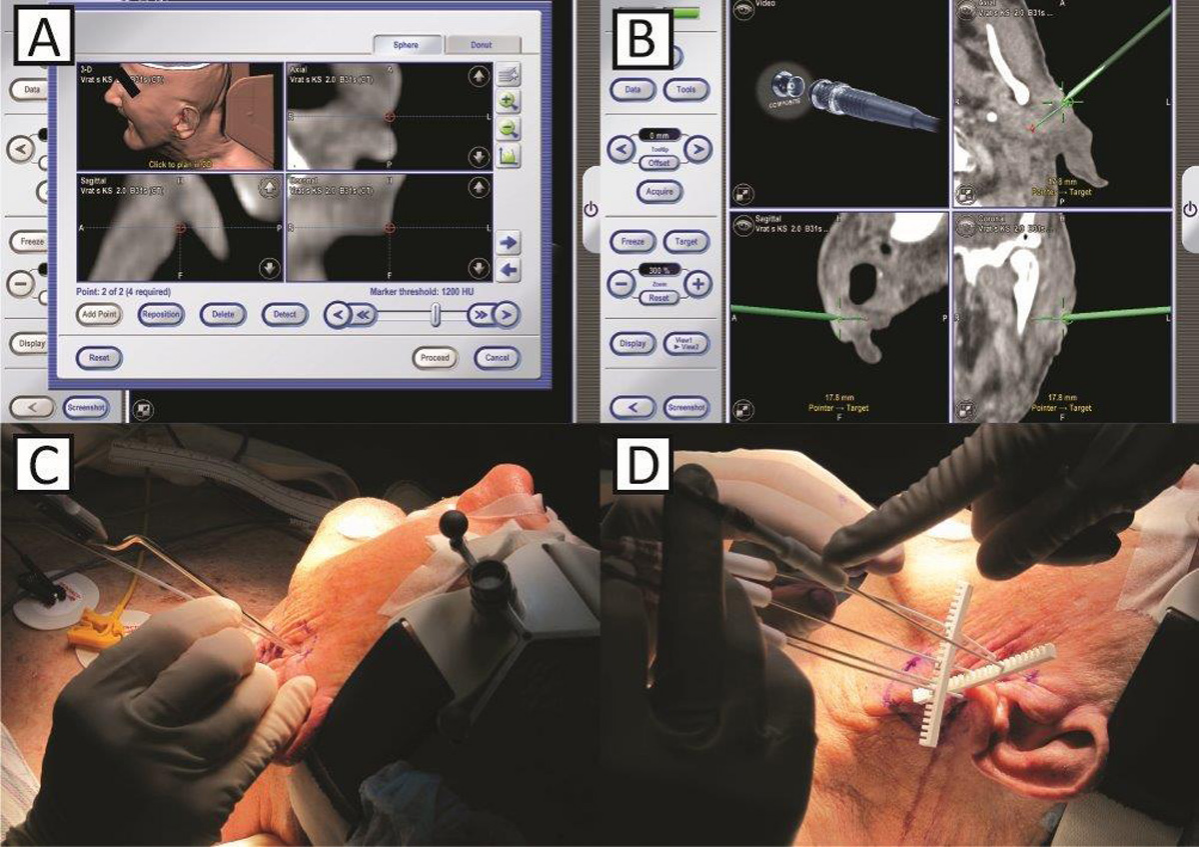
**Treatment 3-D planning for registration and navigation (A)**. Navigation was used to accurately access the planned skin entry points and direction of electrodes (B). Positioning of navigation system needle and first needle electrode (C) and the final positioning of all five needle electrodes (D).

The registration was carried out using standard multipoint technique of predefined points around target surface. Five points adjacent to tumor site were used and good accuracy with error margin towards the slice thickness of CT scan was achieved. The registration star was fixed on patient's head using standard headband (Headband Brainlab AG, Feldkirchen, Germany). Plane of registration star was positioned at an angle to achieve optimum link with infra-red (IR) camera. Plan of work was similar to stereotaxy achieving optimum trajectory from entry point (skin) to target point (tumor or predetermined position or electrodes). Hence the electrodes were positioned according to plan using the virtual line, extending from the tip of the guiding instrument towards the tissue (Figure 4).

Based on the treatment plan, five stainless steel needle electrodes were positioned with navigation system guidance. Electrodes were positioned in a star pattern with 1 cm center-to-center distances between the central electrode positioned in the tumor and the outside electrodes and 1.4 cm center-to-center distance between the outside electrodes; 1000 V was applied between center electrode and the outside electrodes, while 1200 V was applied between each pair of outer electrodes. This was enough to ensure at least 500 V/cm of electric field strength in the whole tumor tissue which is well above reversible threshold for tumor tissue and increases robustness of the treatment.

### Treatment procedure

**Figure 5:**
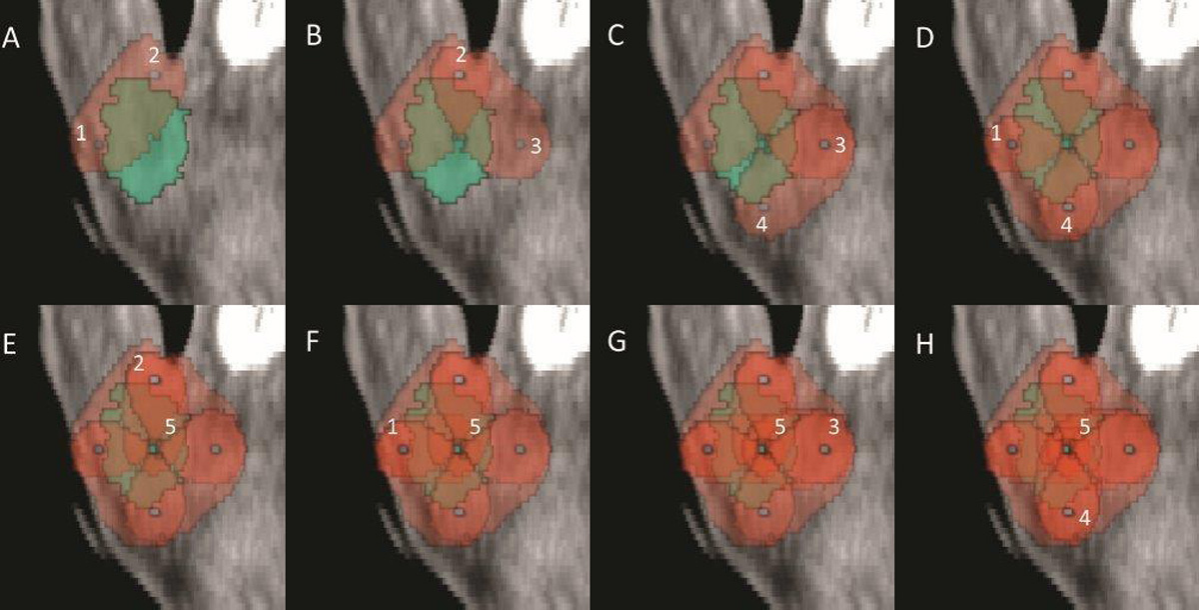
**Progression of metastasis coverage with electric fields in excess of 400 V/cm for the metastasis No. 1**. Each figure (A-H) shows the active electrodes, marked in Arabic numerals, of the 8 electrode pairs used with the coverage following the actual pulse delivery. The figure shows overlapping coverage in most parts of the metastasis, which ensures additional robustness of the treatment. Metastasis is marked light green, and the area exposed to an electric field higher than 400 V/cm appears red. While overlapping coverage with more pulses would eventually lead to irreversible electroporation at the electric fields delivered in this case, it should be noted that only 64 pulses were delivered in total, while typically 90 pulses per electrode pair are used in irreversible electroporation.

The patient was treated under general anesthesia. Five long needle electrodes with 3 cm active tip were used in both metastases [12,15,30]. Firstly, electrodes were positioned in the metastasis No.1 with the help of navigation system and in accordance with patient specific treatment plan. Then, 27.000 IU bleomycin (15.000 IU/m^2^, Heinrich Mack Nachf. GmbH & CO. KG, Illertissen, Germany) was intravenously administered in bolus and after 8 minutes the metastasis No.1 was treated. Eight electric pulses, each in duration of 100 µs, were delivered to each pair of electrodes consecutively by electric pulse generator Cliniporator Vitae^® ^(IGEA, Carpi, Italy) (Figure 5). Immediately after the treatment of the first metastasis, the electrodes were repositioned into metastasis No. 2, which was then treated with the same treatment parameters (total of 6 minutes between the two treatments, i.e. 14 minutes after bleomycin injection). The delivery of electric pulses was synchronized with the absolute refractory period of the heart to additionally ensure the safety of the patient and avoid delivery of pulses during vulnerable period of ventricles [31].

## Results and discussion

### Treatment and clinical outcome

**Figure 6:**
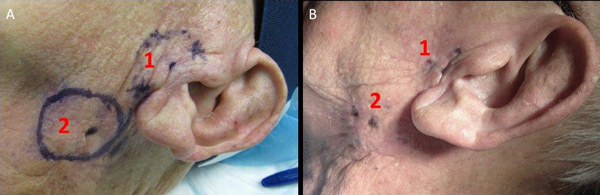
**Patient before (A) and 5 weeks after electrochemotherapy (B)**. Metastases No.1 (1) and No.2 (2) are marked.

**Figure 7:**
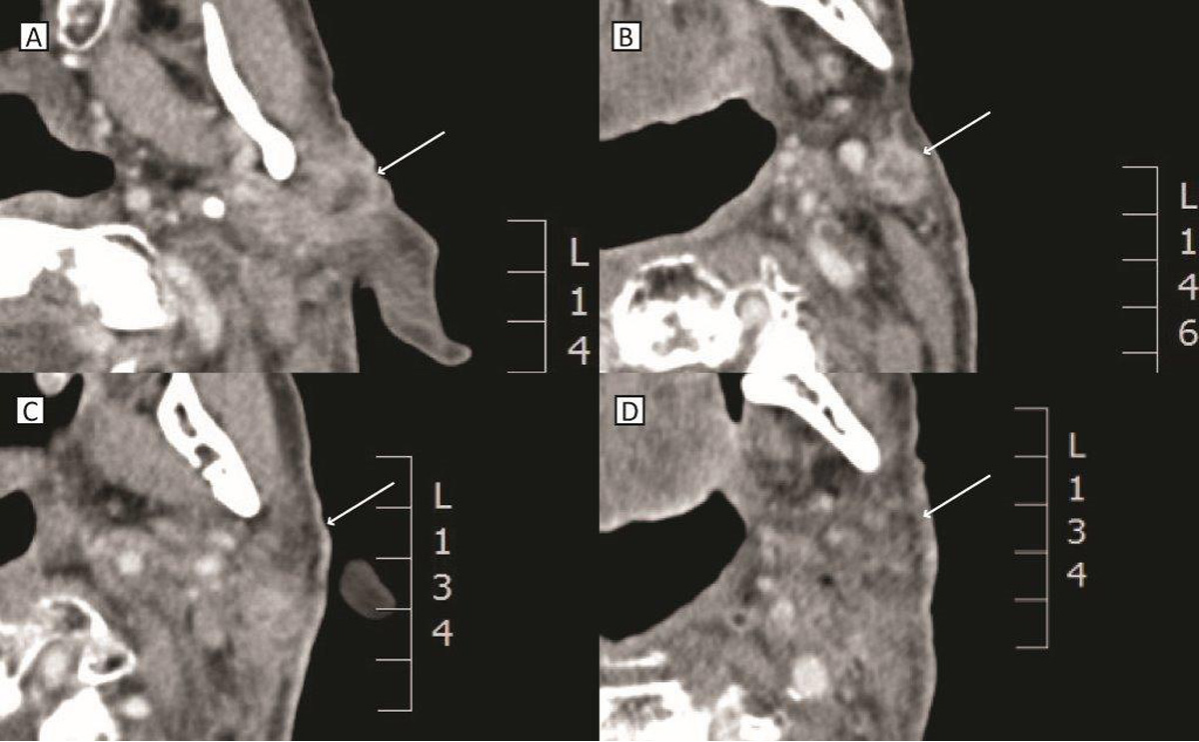
**CT images before (A, B) and after (C, D) electrochemotherapy**. PR of the metastasis No. 1 in the left parotid gland (A, C). CR of the metastasis No. 2 behind the left mandibular angle (B, D). Locations of the metastases are marked with arrows.

Two squamous cells carcinoma lymph-node metastases were treated by electrochemotherapy. The patient presented with the first metastasis of 20.3 mm in diameter sited in the left parotid gland close to the facial nerve (No 1). The second metastasis was located behind the left mandibular angle, in close proximity to the left internal jugular vein, and was 20.6 mm in diameter (No 2) (Figure 4, 6). Electrochemotherapy was performed in general anesthesia, after intravenous injection of bleomycin using single long needle electrodes that can be inserted individually, with 3 cm active tip. The procedure of electrode insertion and execution of electrochemotherapy was successful and without intra- or postoperative complications (e.g. bleeding, edema). The total treatment time took 60 minutes and was completed 14 minutes after bleomycin injection. Thus, both metastases were treated within the generally accepted time frame of 8-28 min after bleomycin injection, as this is considered to be the time when the concentration of bleomycin in the tumor is the highest [32]. The delivery of electric pulses was successful, and no disturbances of the heart rhythm were detected. The patient developed mild facial weakness, which resolved in five hours after procedure. The patient reported only mild pain that lasted 1 week after the treatment and was well controlled by paracetamol. The CT taken 5 weeks after electrochemotherapy demonstrated good antitumor effect (Figure 7). According to RECIST criteria (version 1.1), complete response of the metastasis behind the left mandibular angle (Metastasis No.2) was recorded, and partial response of the metastasis in the left parotid gland (Metastasis No. 1; Figure 6). Six weeks after electrochemotherapy, fine-needle aspiration biopsy of the treated area revealed necrosis and inflammatory cells, without any viable tumor cells in the specimens from both of the treated lesions. In addition, good cosmetic effect was obtained.

### Advantages of the single long needle electrodes

So far, the technology of electrochemotherapy has evolved predominantly for the treatment of cutaneous and superficial tumors, not extending deeper than few cm below the skin. Electrodes with fixed geometry that can reach up to 3 cm in depth are used for this purpose. However, these are often not long enough to reach the base of deep-seated lesions in the head and neck region, compromising treatment results [13]. In addition to that, due to the irregularity in shape of head and neck tumors, the coverage of the whole tumor with such electrodes is often hampered or even not possible. In the future, the development of new types of electrodes may be anticipated, specifically for the tumors in head and neck region. For example, the first prototype has already been designed for the treatment of brain tumors. The electrodes are inserted through the skull, and afterwards extended in umbrella like fashion to encompass the tumors [33].

However, complex anatomy with proximity of several vital structures (e.g. blood vessels, cranial nerves), limited space, bony structures, and usually rather large tumors of irregular shape represent a considerable challenge which could be overcome by using single long needle electrodes. Long needle electrodes were developed for electrochemotherapy of the deep seated tumors. So far, they were used in the treatment of sarcomas and of liver metastases [28,34]. Our group has gained experience in the use of such electrodes for the treatment of liver metastases of the colorectal adenocarcinoma [16]. The recently published study provided evidence that the electrodes with 3 or 4 cm of active, un-insulated part can be placed into the tissue, exposing the tumor to the active part and shielding the normal tissue. Furthermore, their use was safe also in the treatment of tumors adjacent to big tumor blood vessels [17]. Comparison between the effectiveness of electrochemotherapy when using electrodes with fixed geometry and the single long needle electrodes demonstrated that the latter provided comparable antitumor effectiveness to those with the fixed geometry [35].

### Importance of the treatment plan for effective electrochemotherapy

For good antitumor effectiveness of electrochemotherapy, two conditions have to be met: sufficient amount of drug molecules present in the tumor, and adequate tumor coverage by the electric field [11,36]. The second prerequisite ensures that membrane permeability in the whole tumor area is sufficiently increased. Electrodes with fixed geometry have pre-set electrical parameters in electric pulse generator to meet the condition of covering the whole tissue volume which is encompassed between the electrodes. When using the long needle electrodes, the electrical parameters have to be adjusted to approximately 1000 V per cm voltage-to-distance ratio according to the specification of the manufacturer. However, with larger and more irregular shaped tumors, it is often difficult to adequately place the electrodes and determine the sequence of the delivery of electric pulses. Principally, one or two electrodes are placed into the center of the tumor, while the remaining 4 or 5 are placed around it into the normal tissue. The electrodes need to be placed parallel, to ensure that the distance between each electrode remains constant along the length of the active region. With treatment planning of electrode positioning and electric field simulation together with well controlled navigation, this restriction could be relaxed somewhat, e.g. to enable access in difficult-to reach location. The pulse sequence is between the central and peripheral electrodes and in-between the peripheral ones (Figure 4). The treatment plan is needed in order to define the position of electrodes and the amplitude between each pair of electrodes, which would guarantee that the whole tumor including a safety margin will be covered with sufficiently high electric field that would enable permeabilization of the cell membranes (Figure 4). Of course, calculation of the anticipated currents has to take in account also the limitations of the clinical device (*i.e*. max current delivered). The advantage of treatment plan is also in predicting the effectiveness of electrochemotherapy in the clinical situations when tumors are close or in-between the blood vessels or adjacent to the bones. In our patient, treatment plan was prepared with the configuration of 5 electrodes per tumor. In each of the two metastases, one electrode was positioned in the center of the tumor and four around the tumor in the normal tissue ensuring sufficient coverage of the tumor and surrounding tissue (i.e. safety margin) with electric field. The currents during the delivery of pulses are monitored and recorded by the pulse generator. The measured currents correspond well with the computed currents (Table 1). The errors (defined as (I_comp _- I_meas_)/I_meas_, were between -19 and +58 %. The Root Mean Square Errors (RMSE) were 2.1 A and 3.7 A for the first and second metastasis, respectively. Some of the discrepancies between the model and measured values could be stemming from the fact that the patient had received high radiation doses to the region, thus changing the tissue dielectric properties. Also, only four homogeneous tissue types were included in the simulation, while the reality is inevitably more complex. The larger RMSE for the second metastasis probably stems from the fact, that the blood vessels in the simulation were fixed, but their actual location during the treatment could have been shifted by the handling of the metastasis during treatment.

**Table 1:** Comparison of measured currents and currents computed using numerical simulations.

		Metastasis 1				Metastasis 2		
**Electrode pairs**	**Voltage [V]**	**Measured [A]**	**Computed [A]**	**Error [%]**	**Voltage [V]**	**Measured [A]**	**Computed [A]**	**Error [%]**

**1 - 2**	1200	21.5	22.6	5%	1200	22.2	18.4	-17%
**1 - 4**	1200	20.7	20.4	-1%	1200	17.6	15.5	-12%
**2 - 3**	1200	17.6	19.5	11%	1200	20.2	17.5	-13%
**3 - 4**	1200	20.9	21.2	1%	1200	17.6	22.2	26%
**2 - 5**	1000	17.8	21.1	19%	1000	17.8	17.1	-4%
**1 - 5**	1000	23.5	22	-6%	1000	17.2	20	16%
**3 - 5**	1000	17.8	20.2	13%	1000	16.1	15.6	-3%
**4 - 5**	1000	16.2	19.5	20%	1000	12.6	19.9	58%

Based on the treatment plan, the electrodes were effectively placed and the treated area adequately electroporated, which was demonstrated by complete response of the metastasis behind the left mandibular angle (Metastasis No. 2) and partial response of the metastasis in the left parotid gland (Metastasis No. 1; maximal diameter before and after electrochemotherapy: 20.3 mm and 14.3 mm). Based on the radiological examination 5 weeks after electrochemotherapy the difference in the response of the two metastases to electrochemotherapy could most likely be ascribed to inadequate drug distribution. Namely, the area of the Metastasis No. 1 has been previously irradiated with 70 Gy, which most likely compromised vasculature in this region. It has been suggested that this problem could be overcome by combining intravenous and intratumoral bleomycin administration [24].

### Aid of the navigation system

Navigation system aids the surgeon in locating the tumor based on the pre-treatment CT or MRI images and is frequently used in surgical interventions in the head and neck region. The use of navigation system enables superior orientation in 3-D space. Data is acquired by the navigational computer consisting of main unit with interface and processor, sensors on navigational antenna with twin infrared cameras and multiple emitters (IR camera), navigation star fixed on patient and navigated pointer free in 3-D environment of patient's skin [37-40].

Based on our first experience, the use of navigation system substantially improves the accuracy of electrode placement. Pre-treatment CT or MRI images are imported into the system and with the aid of the fixed markers, a precise position of the tumor in the patient can be verified with these images. In addition, the system significantly contributes to the accurate positioning of the electrodes in the patient. This is of crucial importance, since the entry and the angle of the electrode insertion can be controlled also in deep-seated tumors. The drawback of the system used in our case is that the depth of the electrode penetration cannot be controlled; however, this obstacle can be compensated by the measurement of the length of the electrode penetration into the tissue which should be adjusted according to the treatment plan.

In the presented case, treatment planning was coupled with navigation system. All electrodes were positioned according to the treatment plan, and electrochemotherapy executed as planned. As can be seen from Figure 3, the electrodes were positioned to within 1-2 pixels of the marked entry trajectory, corresponding to an error of up to 1.14 mm (the pixel size was 0.57 mm). The depth of the electrode insertion was measured by the ruler (the depth of electrode insertion could not be controlled by the navigation system as the geometry of the needle electrodes and appropriate navigation markers for electrochemotherapy have not yet been implemented into the guidance system). The insertion of the electrodes required some extra time, compared to the routine electrochemotherapy using fixed geometry electrodes. However, one must bear in mind that this technological approach is amenable for specific clinical situations with very limited treatment options that demand more attention than usual cases. In addition, it is expected to improve the efficacy of electrochemotherapy in head and neck region. Namely, currently available data indicate considerably lower efficacy of electrochemotherapy in non-melanoma head and neck cancers, compared to the basal cell carcinoma in the same region (CR rate: 25% vs. 78%) [24,41]. Electrochemotherapy is also very effective in some other tumor types, such as melanoma, with complete response rate of 73.7% after single treatment [42,43]. The reason for this difference is probably the lack of adequate technological solutions, *i.e*. the appropriate electrodes and of the approach, assuring satisfactory coverage of tumor area with electric field. The approach described here may provide improvement of results in such cases, and increase the efficacy of electrochemotherapy with single needles compared to treatment with standard electrodes of fixed geometry. Furthermore, with this technological solution, also deep-seated tumors of the head and neck can be placed on the list of indications for electrochemotherapy.

## Conclusion

Our study is the first showing that coupling treatment planning with the navigation system for precise placement of long single needle electrodes is a feasible and effective way to approach deep-seated tumors in the complex anatomical region of the head and neck. This technological approach may lead to improved therapeutic effectiveness of electrochemotherapy in larger tumors of head and neck region, which are often located deep under the skin, are irregular in shape, and/or close to sensitive structures. Although primarily developed for neurosurgical application, intraoperative navigation has gained acceptance in head and neck surgery, especially in the functional endoscopic sinus surgery. Therefore, the aid of navigation system represents a technological advancement for electrochemotherapy of deep seated tumors, since navigation system can provide identification of tumor position and accurate placement of the electrodes.

## Authors' contributions

**Conception and design: **A Groselj and G Sersa

**Development of methodology: **A Groselj, B Kos, D Miklavcic, J Urbancic

**Acquisition of the data (provided animals acquired and managed patients, provided facilities, etc.): **A Groselj, B Veberic, M Bosnjak, B Kos, M Cemazar, G Kragelj

**Analysis and interpretation of data (e.g. statistical analysis, biostatistics, computational analysis): **B Kos, M Bosnjak, D Miklavcic, A Groselj

**Writing/review and/or revision of the manuscript: **A Groselj, M Cemazar, M Bosnjak, P Strojan, B Kos, D Miklavcic, J Urbancic, G Sersa

**Study supervision: **A Groselj, G Sersa

## Competing interests

All authors declare no conflict of interest, except Damijan Miklavcic, who holds patents on electrochemotherapy that have been licensed to IGEA S.p.a. and is also a consultant to IGEA.

## Declarations

The authors acknowledge the financial support from the state budget by the Slovenian Research Agency (program no. P3-0003, P2-0249, P3-0307). Costs for publication were funded by program grant P3-0003. Research was conducted in the scope of EBAM European Associated Laboratory (LEA). This manuscript is a result of the networking efforts of the COST Action TD1104 (http://www.electroporation.net). We are thankful to prof. Greta Strojan Flezar, for the cytopathology of the tumor specimens.

This article has been published as part of *BioMedical Engineering OnLine *Volume 14 Supplement 3, 2015: Select articles from the 6th European Conference of the International Federation for Medical and Biological Engineering (MBEC 2014). The full contents of the supplement are available online at http://www.biomedical-engineering-online.com/supplements/14/S3.
